# Developing patient-friendly genetic and genomic test reports: formats to promote patient engagement and understanding

**DOI:** 10.1186/s13073-014-0058-6

**Published:** 2014-07-31

**Authors:** Susanne B Haga, Rachel Mills, Kathryn I Pollak, Catherine Rehder, Adam H Buchanan, Isaac M Lipkus, Jennifer H Crow, Michael Datto

**Affiliations:** Center for Applied Genomics, Duke University School of Medicine Center for Personalized and Precision Medicine, Duke University Health System, 304 Research Drive, Durham, NC 27708 USA; Cancer Control and Population Sciences, Duke Cancer Institute, 2424 Erwin Rd, Durham, NC 27705 USA; Community and Family Medicine, Duke University Medical Center, 318 Hanes House, DUMC 2914, Durham, NC 27710 USA; Duke University Health System Clinical Laboratories, 4425 Ben Franklin Blvd, Durham, NC 27704 USA; Duke School of Nursing, 307 Trent Dr, Durham, NC 27710 USA

## Abstract

With the emergence of electronic medical records and patient portals, patients are increasingly able to access their health records, including laboratory reports. However, laboratory reports are usually written for clinicians rather than patients, who may not understand much of the information in the report. While several professional guidelines define the content of test reports, there are no guidelines to inform the development of a patient-friendly laboratory report. In this Opinion, we consider patient barriers to comprehension of lab results and suggest several options to reformat the lab report to promote understanding of test results and their significance to patient care, and to reduce patient anxiety and confusion. In particular, patients’ health literacy, genetic literacy, e-health literacy and risk perception may influence their overall understanding of lab results and affect patient care. We propose four options to reformat lab reports: 1) inclusion of an interpretive summary section, 2) a summary letter to accompany the lab report, 3) development of a patient user guide to be provided with the report, and 4) a completely revised patient-friendly report. The complexity of genetic and genomic test reports poses a major challenge to patient understanding that warrants the development of a report more appropriate for patients.

## Introduction

Genetic and genomic testing is expected to grow substantially, shifting from esoteric to routine laboratory testing [1,2]. It is estimated that more than $5 billion is currently spent on genetic and genomic testing in the United States, and this is projected to increase to $25 billion by 2021 [3]. Furthermore, the complexity of testing will increase with the expanded use of comprehensive genomic testing platforms such as whole genome sequencing and chromosomal microarray testing [4]. Concurrent with advances in molecular testing, patient access to lab reports is increasingly available through patient health portals and electronic medical records (EMRs). Greater access to test results may help to promote more effective and satisfactory patient-provider engagement, patient understanding, shared decision-making, and adherence with clinical recommendations [5–16]. However, a lack of patient comprehension of genetic and genomic information may substantially limit the benefits of patient access to their reports, as has been shown with other types of medical information [17–19]. For example, patients who do not understand their health information may show lower engagement and satisfaction with their care [20], display poor adherence to recommended interventions [21–25], and experience anxiety [13]. Furthermore, accessing genetic or genomic lab reports may increase patient anxiety due to confusion about the purpose and outcomes of testing [26], and thus adversely influence their medical care.

Little guidance is available on how best to present genetic or genomic lab results in an understandable format and at an appropriate reading level for patients. With respect to health information in general, Tang *et al.* [18] recommend that it must be presented ‘in ways that enable the individual to understand and to act on the information contained in the record’. Although the readability of test reports has not been specifically evaluated (understandably so, given that they have not been intended for patient use), analysis of health educational materials intended for patients shows that many are not suitably written for patients, highlighting the challenges of writing for a lay audience [27–29]. Several ongoing efforts have evaluated the content of reports from genetic testing, particularly for clinical genome sequencing [30–32]. However, these recommendations are generally focused on the needs of the clinician, although patient preferences were recognized as an important element in the reporting of incidental findings [31].

With increased patient access to lab reports, now is the time to consider the development of a patient-friendly report format to improve patient comprehension and engagement. In addition, as more and more non-geneticist providers with varying levels of knowledge and experience are ordering genetic and genomic testing, a patient-friendly reporting format may also benefit clinicians. Early use of whole genome sequencing indicates that the time required for counseling is substantial, with reports of post-test sessions of 2 to 3 hours [33–36]; a patient-friendly lab report may help reduce the time needed to explain test results, leaving more time instead to discuss the significance of the results for health care, and may increase the use and feasibility of testing. In this Opinion, we review the current reporting formats, discuss potential patient barriers to comprehension, and propose several options to reformat genetic and genomic lab reports to promote patient comprehension and engagement.

## Current genetic and genomic test reporting formats

In the United States, the content and availability of test result reports are regulated by federal regulations (42 CFR 493.1291) and professional guidelines [37–44]. The lab report must include information about the patient, ordering physician, specimen collection, test method, results and interpretation in a format and language understandable to a general practice physician (Box 1). Overall, genetic and genomic lab reports tend to be very technical in nature due to the complex testing platforms used and variable test outcomes. For example, reporting genetic variants may include information about the gene (such as gene name, zygosity, cDNA nomenclature, rs number) and protein (protein nomenclature), as well as the phenotype or result interpretation (for example, poor metabolizer) [45]. Clinical genome sequencing lab reports may differ somewhat from more traditional, single gene test results given the comprehensive nature of sequencing. Current guidelines suggest prioritization of test results based on the patient’s phenotype, determination of which variants are included in the report (for example, optional inclusion for variants with no known disease association), and reporting of incidental findings [32,46].

Although the content of reports is regulated, the format and presentation are not, and are largely decided by the lab performing the test. In some reports, a summary statement of the results is included in the section following the patient and provider information at the top of the report. The summary statement may start with the words ‘positive’ or ‘negative’, followed by a description of the specific genetic change identified as a likely cause for the patient’s condition (for example, ‘Positive - an established cause of the reported phenotype was identified’). Additional information, such as the biochemical, molecular or cellular effect of the change may then be included in an expanded results section. Regarding the interpretation and follow-up sections, the scope of information provided by different labs can also vary dramatically. Some labs simply list the acquired mutations using standard nomenclature and defer all interpretation to the care provider receiving the result. Other labs provide references and treatment recommendations based on the mutations identified. In all but a few, very rare cases, reports are written and formatted for health providers.

This heterogeneity of reporting formats may influence clinical interpretation, given the differing levels of familiarity with genetic and genomic tests among ordering clinicians [47–51]. Studies of health providers’ satisfaction and perception of the utility of clinical lab reports identified several areas for improvement with respect to the content, use of ambiguous terminology, complexity of results, unclear interpretation of results, and lack of follow-up recommendations [30,52–55]. In addition, the common use of scientific or medical jargon to describe the testing methodology, results (for example, heterozygous mutation, conserved splice site, splicing, alleles, missense mutation), interpretation (for example, ‘this mutation most likely disrupts a conserved splice site and abrogates normal splicing, causing severe defects in LMNA protein production’) and follow-up (for example, ‘maternal metaphase FISH analysis utilizing interval specific BAC probes are recommended to investigate the suspected familial rearrangement’) may pose challenges to comprehension for both providers and patients.

Until recently, genetic test results were most often verbally communicated by a clinician or genetic counselor [56–61], and the patient did not receive a copy of the report unless requested. However, US health information technology regulations currently require patient electronic access to their records (Stage 2, Core Measure 7) [62,63], and many health systems have implemented enterprise-wide EMR systems that have a fully functional patient portal enabling access to documents including lab reports [64]. Specifically, for a clinical or health organization to meet current meaningful use requirements for EMRs, 50% of all patients much be given timely access (within 4 days) to their health information after their clinical encounter, including lab results. Some clinicians may have exceptions for information that can only be released to the EMR after the patient has been contacted by their provider (after a 4-day window). Genetic testing may be considered a type of information that is too complex to release directly to the patient without interpretation by a clinician or geneticist first. Alternatively, patients may request a copy of the report directly from the testing lab [65].

## Patient barriers to comprehension of genetic and genomic test reports

While several studies have explored the communication of genetic test results to patients, fewer studies have considered the patient experience of the actual lab report [26]. Important predictors of patients’ experiences include their understanding of the purpose and outcomes of testing, and how the results may impact diagnosis or medical management, their satisfaction and, ultimately, health outcomes [66,67]. We describe several barriers that may pose challenges to patient comprehension of genetic and genomic test reports, including health literacy, genetic literacy, e-health literacy (for results reported via an online patient portal) and risk perception.

Health literacy is broadly defined as ‘a constellation of skills, including the ability to perform basic reading and numerical tasks required to function in the health care environment’ [68]. Low health literacy may affect the ability of patients to comprehend and/or utilize genomic risk information to reduce disease risk and participate in healthcare decisions [69–71]. The ‘readability gap’- the disparity between the language used in health documents and the reading level of the user - has been reported to be a major barrier to patient comprehension [72–77]. In addition, low numeracy skills have been associated with worse perceived self-efficacy and self-management behaviors [78], and contribute to health disparities [79]. As many genetic and genomic tests may include a numeric risk or probability of developing a given phenotype, communication and comprehension of risk will be a major challenge for many health providers, including genetic counselors [80] and patients, respectively. Presentation of risk in multiple formats (text, numerically, pictorially) [81] and assessment of patient comprehension through teach-back techniques are often recommended [82]. Novel technologies such as avatars and personalized graphics are also being explored to improve patient comprehension [83].

Genetic literacy has been defined as ‘sufficient knowledge and appreciation of genetics principles to allow informed decision-making for personal well-being’ [84]. Unfamiliarity with genetic concepts and terms may pose barriers to understanding of or appropriately acting upon genetic test results [85–87]. For example, unfamiliarity with gene nomenclature, symbols denoting alleles (for instance, *1), and genetic terminology (such as carrier, heterozygosity) will likely exacerbate patient confusion. The use of genetic terminology or jargon may also pose challenges for providers and it has been recommended that reports be written in an understandable language for non-geneticist health providers [44,88].

As more health systems transition to EMRs with patient portals, e-health literacy may pose an additional challenge for patients. E-health literacy refers to the ability to ‘seek, find, understand, and appraise health information from electronic sources and apply the knowledge gained to addressing or solving a health problem’ [89]. In general, patient experiences with EMRs [90] and online health communication [91] have been reported to be positive. Accessing health information online is convenient and enables the patient to control the pace and amount of information consumed [92]. Interactive computer programs have also been found to be more effective than standard genetic counseling for increasing knowledge of genetic testing [93,94]. Comfort with online genetics communication has been associated with previous online health information seeking, a healthy lifestyle and a positive attitude towards genetics [95]. However, limitations in e-health literacy may affect a patient’s likelihood to access online resources and lab reports through a health portal.

In addition to the three literacy challenges (health literacy, genetic literacy and e-health literacy), patients’ understanding of the actual test results may be affected by their perception of risk [96,97], which is based on a combination of factors, including their motivational and emotional state, underlying expectations, and family members’ experience with an illness [98–101]. For example, lifetime risk estimates based on a positive genetic test result (a causative mutation is detected) do not appear to substantially influence risk perception compared to risk estimates based on family history [102,103]. However, risk estimates based on a negative test result (a disease-causing genetic mutation is not detected) may lead to differences in risk perception compared to risk estimates based on family history [102,103]. The ‘meaning’ or implication of a test result is further framed by ethnicity and culture [104]. Many health behavior models emphasize the importance of patients’ perception of risks [105]; thus, an accurate understanding of their test result may encourage patients to revise these perceptions, which may lead to improved compliance with clinical follow-up or medications [106,107].

## Moving towards a patient-friendly test reporting format

The development of patient-friendly lab test reports will require an understanding not only of what types of information patients desire to learn and need to understand to interpret the test result, but also what information will be most helpful in influencing the appropriate utilization of results [6] and how best to present that information to optimize patient comprehension. However, few studies have explored patients’ needs with respect to the development of patient-friendly health records [17,20], and only one study has described efforts to develop a patient-friendly pathology report [108]. Keselman *et al.* [17] reported that patients desired help to understand the purpose of the test, the reporting range or units of the results reported, and the interpretation of results. Elder and Barney [20] reported that patients’ understanding of the purpose of testing, the actual lab results (with a description of desired values), and the significance of the result for their care was critical to their satisfaction.

An important consideration when revising lab reports is the amount of education and information given to patients prior to testing. Pre-test communication is an important part of the overall testing process, as it prepares patients for the type of possible results from genetic or genomic testing (including inconclusive results and incidental findings) and how results may be used to inform their care. As a result, pre-test communication can reduce the time and need to provide detailed background information about testing when communicating results or in the lab report. However, due to the lack of standard protocols or accepted patient educational materials for genetic and genomic testing, and depending on the provider and their access to genetics experts, the extent of pre-test communication provision may vary widely. In addition, while many different patient resources have been developed to promote awareness and inform decision-making, particularly for cancer susceptibility testing [109], disparities between patients’ reading level and the resources continue to pose major challenges [110]. Labs could encourage pre-test education by requiring signed patient consent forms or providing educational materials with marketing materials or lab requisition forms.

Lab professionals can serve as an integral part of the healthcare team by promoting effective communication with patients via their reports. The identification and curation of reliable information to aid in the comprehension of test results is an essential function of clinical lab professionals. This responsibility can no longer be deferred to the ordering physician. An understandable lab report will prepare the patient to engage in meaningful discussions with their provider about their care, optimizing limited time and healthcare resources. Many of the principles applied to the development of current lab reports for clinicians could be applied to the development of patient-friendly lab reports, including synoptic reporting frameworks (or partitioning the report into uniform, easy-to-find sections) [52] and patient narratives [53].

**Figure 1:**
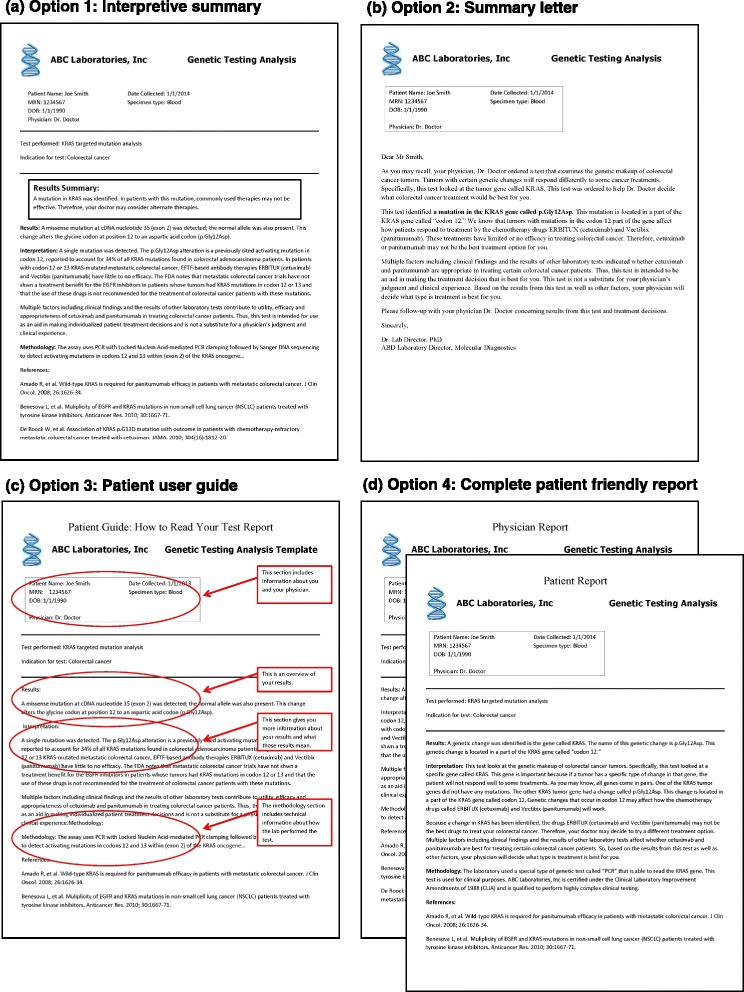
**Mock-up reports of the four proposed options for the pharmacogenetic mutation analysis test of the** ***KRAS*** **gene to inform treatment decisions for colorectal cancer patients [** 111 **-** 113 **]** **. (a)** Option 1: test report with interpretative summary box written in patient-friendly language describing the test, test result, and interpretation. **(b)** Option 2: summary letter with more extensive description of the test and test result in patient-friendly language. **(c)** Option 3: patient user guide to accompany the test report to help patients navigate and understand sections of the test report. **(d)** Option 4: first page of a completely patient-friendly lab test report written in patient-friendly language. Please note that these sample reports are not actual clinical reports. Note, for option 2, that the Flesch-Kincaid Reading Ease [114] score is 51 (on a scale from 1 to 100), corresponding to a 9th grade reading level. For option 4, the Flesch-Kincaid Reading Ease score is 56 (on a scale from 1 to 100), corresponding to a 9th grade reading level. However, if the names of the drugs are removed, it decreases to a 7th to 8th grade reading level.

On the basis of a review of the literature and of our collective experiences, we propose four possible options to improve genetic and genomic lab reports. Each of the proposed options is intended to address the patient barriers of health literacy, genetic literacy and e-health literacy and can be adopted for paper or electronic reports to accommodate differing health systems and patient preferences. Figure 1 provides a mock-up of each of the options for the pharmacogenetic test for *KRAS* mutation analysis. Table 1 includes an overview of the benefits and challenges of each recommended option.

**Table 1:** **The benefits and challenges of reformatting lab reports to address patient barriers to comprehension**

**Option**	**Benefits**	**Challenges/limitations**
Option 1: Interpretive summary	· Includes short patient-friendly summary of test performed, results, interpretation and clinical recommendations	· Only part of report that is patient-friendly is the boxed summary
	· Easy for patients to find important results in multi-page report	
	· Requires minimal effort from lab	
	· May aid providers as well as patients in understanding results	
	· Clinicians can ‘borrow’ text from summary box to include in EMR	
Option 2: Summary letter	· Includes an overview of the information in the results report in a patient-friendly letter	· Would require an EMR to work most effectively
	· There is already an established practice in place (for example, genetic counselors writing letters)	· Requires an extensive amount of time to set up templates/standardized text
	· Letters are personalized for each patient	· Requires additional time for each patient
		· Requires collaboration between lab and clinician to write detailed letter
Option 3: Patient user guide	· Includes an explanation of each section of the report in patient-friendly language	· Would benefit from having an EMR in place
	· Can assist patients in use of patient portal to review results	· Requires an extensive amount of time to set up templates
	· Providing user guide prior to testing may enable patients to be prepared for result	· Only text that will be patient-friendly is the guide; text within the report may still be difficult to understand
	· Would require minimal effort for each individual patient	
Option 4: Complete patient-friendly report	· The entire report is written in patient-friendly language	· Requires an extensive amount of time to set up templates/standardized text
	· May aid providers as well as patients in understanding results	
	· Providing a patient-friendly report in addition to a standard report may satisfy patients with higher literacy	

### Option 1: Interpretive summary

Since the test result/finding is the crux of the report, one format option is to add a boxed results summary section (‘interpretive summary’) on the first page for patients, while keeping the remainder of the report in its current format. Prominently displaying key information in a language that is understandable can help patients to identify more easily the section(s) of the report of interest to them and reduce frustration. The American College of Medical Genetics and Genomics (ACMG) has recommended that a ‘succinct, high-level interpretive result should be provided at the start of the interpretation’ for testing results from next-generation sequencing [32]. However, the proposed ACMG summary is intended for providers and we suggest that a simple, concise interpretive summary written in plain language for patients could include a brief description of the test performed, test result, result interpretation and clinical recommendations [26,91]. With this option, the patient can immediately view his or her results, without wading through a multi-page report. Additionally, clinicians may choose to incorporate the interpretive summary text in their encounter note within the EMR, to which patients may have access. The content and format of the summary could be standardized to promote familiarity and ease of use for patients, regardless of the type of test performed. The corresponding section in the lab report could be referenced in the interpretive summary to direct patients to specific parts of the lab report for more information (for example, ‘see Section Option 1: Interpretive summary’). Overall, the effort required to add a single new section would be minimal, less disruptive for clinicians as the rest of the report would remain the same, and would facilitate patients’ ability to quickly locate information of greatest interest while also continuing to provide access to the full report for patients interested in additional information.

### Option 2: Summary letter

Similar to an interpretive summary, a summary letter or patient letter could be appended to the clinical lab report, providing more extensive information about testing and the test result. In common with some other healthcare providers, genetic counselors typically send a letter summarizing the test result and subsequent discussion in the counseling session [115], but no data are available regarding how prevalent the practice is or if letters are only sent for certain results (for instance, if letters are only sent to patients with new diagnoses). Labs could prepare a summary letter intended for the patient that would be sent with the test report; the provider may have the option to add information about interpretation and follow-up, and could then forward the letter to the patient or upload it to the patient’s EMR. A shared EMR, such as between a lab and a clinician within a hospital or academic medical center, would enable providers to insert additional information to contextualize the results before sending the letter to the patient. The template of the patient letter could include a description of the purpose of the test, the test result, and any follow-up recommendations (for example, follow-up office visit, additional testing) [115–117]. For test results with potential psychological effects, a section on coping-related strategies could also be included [115,118]. The primary challenge will be to ensure that letters are written in a language understandable to the patient [119,120], with limited use of medical jargon or concepts [121,122]. This approach is being used to communicate pharmacogenetic test results to patients in a study at St Jude Children’s Research Hospital; a letter communicating the child’s test results is sent to parents along with an information fact sheet [123], and the patient’s specific findings and recommendations are inserted into a letter template using patient-friendly language. Use of a patient letter could supplement the patient-provider discussion of test results and serve as a written record of testing that patients may share with other providers or family members.

### Option 3: Patient user guide

To assist patients with navigating a genetic or genomic test report, which can often span several pages, a ‘user guide’ could be developed (for example, ‘How to Read Your Test Report’). As often provided for a range of consumer products, the user guide could give a general explanation of each section of the test report. The user guide would resemble a test report and include an explanation of each section in patient-friendly language with a sidebar or call-outs (for example, ‘this section describes how the test was performed’). If the format of the test report was standardized, a single user guide could be developed and sent with each lab report, thereby substantially reducing the effort required by the lab to develop the guide. A copy of the user guide could also potentially be given to the patient when testing is ordered, during pre-test communication, to familiarize and prepare patients on how to read the lab report. To assist patients with limited e-health literacy, a printed user guide could provide tips on how to use online tools provided in the electronic version of the report or provide instructions on how to access the lab report through the lab’s website, if available. Additionally, for electronic lab reports, the user guide could be integrated into the online report; for example, each section heading of the report could be linked to or have a pop-up explanation of that section. Patient understanding of either paper or electronic lab reports could be further improved with auxiliary learning aides, explanations, definitions or links to additional resources [124,125], appended or integrated into the lab report. For example, the US Centers for Disease Control and Prevention recommends that if no mutation is detected, the report should state ‘no mutation detected’ rather than ‘normal’ [44]. However, patients may interpret this finding as a negative result, when in fact it may just rule out one possible gene for a genetically heterogeneous disease, or rule out one of many possible differential diagnoses. Therefore, providing a detailed description of the meaning and implication of the result that is understandable to the patient is important. The US National Human Genome Research Institute has developed a glossary that could be linked to the test report or included in the user guide [126]. By directing patients to specific written or online resources, patients will avoid the additional challenge of identifying credible resources to decipher the information within the lab report.

### Option 4: Complete patient-friendly report

Developing a patient-friendly version of the entire report is a fourth option that would probably provide patients with the most information, but would require extensive work for labs to develop one for each test. The format of the report, including section headings such as results, methodology and interpretation, may remain the same but the text or figures in each section would need to be revised to be more understandable to patients. Clinicians (as well as some patients) may still desire to have the detailed, more formal report and, therefore, both a patient and standard version of the test report may need to be available. Sending two versions of a test report may be preferred by patients of high health literacy or educational status, who may view the patient-friendly version as less credible or unable to meet their informational needs. Like the patient letter option, a completely revised patient-friendly report could serve as a record for patients to share with other providers and family members.

### Other options and considerations

We have presented just four possible options for improving the format of genetic and genomic lab test results for the benefit of patients. There are undoubtedly other possible formats, as well as educational aides (for example, videos), that could be used to promote patient comprehension that are not discussed here. With the increased use of EMR and online portals, there are even greater possibilities for improvement, such as enhancing interface design to include links to additional information (for example, glossary, other websites) to promote comprehension and usability [17,127–129]. Using an online system for reviewing results may also present the opportunity to create interactive reports that will further engage the patient and potentially promote increased understanding and recall.

Currently, lab reports for genetic and genomic testing are primarily text based, and are written in a language beyond the reading level of most patients. All of the proposed options will require the use of plain language or consumer health vocabularies [17,128,130] to achieve the 6th to 7th grade reading level recommended for patient health materials [129] and minimize potential misinterpretation of results. Additionally, presenting results in numeric and non-numeric text [131], as well as in graphical modes, may also improve patient comprehension [132,133]. Tables may be challenging for some patients to interpret [134] and a summary or synopsis may be helpful to explain the significance of the information for the patient.

The burden on labs to reformat test reports must also be considered. Lab expenses related to re-formatting of reports, information technology and increased patient engagement from requests for results may be recovered through preparatory fees [19] or clinical consultation fees for patients requiring further assistance to understand the lab report (such as a phone consultation). Prior to the broad implementation of patient-friendly lab reports, assessments of providers’ preferences for a single lab report or separate reports (patient and provider) as well as the impact of the patient-friendly report on practices will also be needed. These considerations will affect which format option will be best for the lab, the clinicians, and the patient population they serve. Each option has unique benefits and challenges to its implementation (Table 1). Of the formats we have proposed, option 1 would probably require the least amount of effort to develop and implement, whereas option 4 would probably require the most. Both options 2 and 3 would probably require a similar degree of effort to develop and implement, although electronic reporting of lab results may facilitate implementation of option 3. Conversely, option 3 would be an independent, stand-alone aid to assist patient navigation of the lab report, whereas options 2 and 4 would need to be individualized for each patient’s result.

## Conclusions

Patients are increasingly able to access their lab reports via EMRs and patient portals or directly upon request from the testing lab. However, patients may have difficulty understanding genetic and genomic lab reports due to low health, genetic or e-health literacy and complexity of the test result, and therefore, may benefit from a re-designed reporting format. We have proposed four options for consideration to reformat genetic and genomic lab reports to promote patient comprehension: inclusion of an interpretive summary, a summary letter, a patient user guide, or a patient-friendly version of the entire report. The proposed formats should be developed using patient-friendly language and include non-text presentations of the results to improve comprehension and utility for patients with varying levels of literacy.

It will be important to find the appropriate balance of meeting patient needs with limiting disruption to provider practice and burden on testing labs. The development of patient-friendly formats would not be impacted by current regulations for lab reporting if the required content is included in the current reporting format for providers or within the patient-friendly version. Before patient-friendly lab reports can be developed, research will be needed to assess patient needs and preferences for which information is included, how it is presented and organized, comprehension, impact on patient behaviors and patient satisfaction. Although the clinicians’ role as interpreter of results and provider of follow-up services will continue to be an integral part of genetic and genomic testing to contextualize the results in light of the individual patient’s clinical history, with patients’ increased access to results, it is important to consider their needs as well.

## Box 1. Current content of molecular test reports [32,44]

• Patient name/date of birth

• Date and (if applicable) time of specimen collection/receipt in lab

• Referring physician/authorized person who ordered test

• Test performed/indication for testing

• Test background

• Test methodology, including nucleic acid targets of test

Test results

○ DNA variants

▪ Individual variant interpretations

▪ Variants of unlikely clinical significance

• Interpretation of test results/summary

• Test limitations

• References to literature (if applicable)

• Recommendations

o Follow-up care/testing

o Genetics consultation (where appropriate)

• Implications of test results for relatives or family members who might benefit (if applicable)

• Statement indicating that test results and interpretation are based on current knowledge and technology

## Abbreviations

ACMG: American College of Medical Genetics and Genomics
EMR: Electronic medical record

## References

[1] The Lewin Group: *Laboratory Medicine: A National Status Report.* ᅟ ᅟ 2008.

[2] Practice BTP (2012). The Economic and Functional Impacts of Genetic and Genomic Clinical Laboratory Testing in the United States.

[3] UnitedHealth (2012). Personalized Medicine: Trends and Prospects for the New Science of Genetic Testing and Molecular Diagnostics.

[4] Miller DT, Adam MP, Aradhya S, Biesecker LG, Brothman AR, Carter NP, Church DM, Crolla JA, Eichler EE, Epstein CJ, Faucett WA, Feuk L, Friedman JM, Hamosh A, Jackson L, Kaminsky EB, Kok K, Krantz ID, Kuhn RM, Lee C, Ostell JM, Rosenberg C, Scherer SW, Spinner NB, Stavropoulos DJ, Tepperberg JH, Thorland EC, Vermeesch JR, Waggoner DJ, Watson MS (2010). Consensus statement: chromosomal microarray is a first-tier clinical diagnostic test for individuals with developmental disabilities or congenital anomalies. Am J Hum Genet.

[5] Baldry M, Cheal C, Fisher B, Gillett M, Huet V (1986). Giving patients their own records in general-practice - experience of patients and staff. Brit Med J.

[6] Fischbach RL, Sionelo-Bayog A, Needle A, Delbanco TL (1980). The patient and practitioner as co-authors of the medical record. Patient Couns Health Educ.

[7] Fisher B, Britten N (1993). Patient access to records: expectations of hospital doctors and experiences of cancer patients. Br J Gen Pract.

[8] Delbanco T, Walker J, Bell SK, Darer JD, Elmore JG, Farag N, Feldman HJ, Mejilla R, Ngo L, Ralston JD, Ross SE, Trivedi N, Vodicka E, Leveille SG (2012). Inviting patients to read their doctors' notes: a quasi-experimental study and a look ahead. Ann Intern Med.

[9] Earnest MA, Ross SE, Wittevrongel L, Moore LA, Lin CT (2004). Use of a patient-accessible electronic medical record in a practice for congestive heart failure: patient and physician experiences. J Am Med Inform Assoc.

[10] Green BB, Cook AJ, Ralston JD, Fishman PA, Catz SL, Carlson J, Carrell D, Tyll L, Larson EB, Thompson RS (2008). Effectiveness of home blood pressure monitoring, Web communication, and pharmacist care on hypertension control: a randomized controlled trial. JAMA.

[11] Nazi KM (2010). Veterans' voices: use of the American Customer Satisfaction Index (ACSI) Survey to identify My HealtheVet personal health record users' characteristics, needs, and preferences. J Am Med Inform Assoc.

[12] Ralston JD, Hirsch IB, Hoath J, Mullen M, Cheadle A, Goldberg HI (2009). Web-based collaborative care for type 2 diabetes: a pilot randomized trial. Diabetes Care.

[13] Woods SS, Schwartz E, Tuepker A, Press NA, Nazi KM, Turvey CL, Nichol WP: **Patient experiences with full electronic access to health records and clinical notes through the My HealtheVet Personal Health Record Pilot: qualitative study.** *J Med Internet Res* 2013, **15:**e65. 10.2196/jmir.2356 PMC3636169 23535584

[14] Zhao J, Grant SF (2011). Advances in whole genome sequencing technology. Curr Pharm Biotechnol.

[15] Tomkins CS, Braid JJ, Williams HC (2004). Do dermatology outpatients value a copy of the letter sent to their general practitioner? In what way and at what cost?. Clin Exp Dermatol.

[16] Treacy K, Elborn JS, Rendall J, Bradley JM (2008). Copying letters to patients with cystic fibrosis (CF): letter content and patient perceptions of benefit. J Cyst Fibros.

[17] Keselman A, Slaughter L, Smith CA, Kim H, Divita G, Browne A, Tsai C, Zeng-Treitler Q (2007). Towards consumer-friendly PHRs: patients' experience with reviewing their health records. AMIA Annu Symp Proc.

[18] Tang PC, Ash JS, Bates DW, Overhage JM, Sands DZ (2006). Personal health records: definitions, benefits, and strategies for overcoming barriers to adoption. J Am Med Inform Assoc.

[19] Young MJ, Scheinberg E, Bursztajn H (2014). Direct-to-patient laboratory test reporting: balancing access with effective clinical communication. JAMA.

[20] Elder NC, Barney K (2012). But what does it mean for me? Primary care patients' communication preferences for test results notification. Jt Comm J Qual Patient Saf.

[21] Dimatteo MR (2004). The role of effective communication with children and their families in fostering adherence to pediatric regimens. Pat Educ Couns.

[22] Martin LR, Williams SL, Haskard KB, Dimatteo MR (2005). The challenge of patient adherence. Ther Clin Risk Manag.

[23] Linn AJ, van Dijk L, Smit EG, Jansen J, van Weert JC (2013). May you never forget what is worth remembering: the relation between recall of medical information and medication adherence in patients with inflammatory bowel disease. J Crohns Colitis.

[24] Dang BN, Westbrook RA, Black WC, Rodriguez-Barradas MC, Giordano TP: **Examining the link between patient satisfaction and adherence to HIV care: a structural equation model.** *PLoS One* 2013, **8:**e54729. 10.1371/journal.pone.0054729 PMC3559888 23382948

[25] Weingarten SR, Stone E, Green A, Pelter M, Nessim S, Huang H, Kristopaitis R (1995). A study of patient satisfaction and adherence to preventive care practice guidelines. Am J Med.

[26] Brewer NT, Richman AR, DeFrank JT, Reyna VF, Carey LA (2012). Improving communication of breast cancer recurrence risk. Breast Cancer Res Treat.

[27] Berland GK, Elliott MN, Morales LS, Algazy JI, Kravitz RL, Broder MS, Kanouse DE, Muñoz JA, Puyol JA, Lara M, Watkins KE, Yang H, McGlynn EA (2001). Health information on the Internet: accessibility, quality, and readability in English and Spanish. JAMA.

[28] Eysenbach G, Powell J, Kuss O, Sa ER (2002). Empirical studies assessing the quality of health information for consumers on the world wide web: a systematic review. JAMA.

[29] Kasabwala K, Agarwal N, Hansberry DR, Baredes S, Eloy JA (2012). Readability assessment of patient education materials from the American Academy of Otolaryngology–Head and Neck Surgery Foundation. Otolaryngol Head Neck Surg.

[30] Scheuner MT, Hilborne L, Brown J, Lubin IM (2012). A report template for molecular genetic tests designed to improve communication between the clinician and laboratory. Genet Test Mol Biomarkers.

[31] Brownstein CA, Beggs AH, Homer N, Merriman B, Yu TW, Flannery KC, Dechene ET, Towne MC, Savage SK, Price EN, Holm IA, Luquette LJ, Lyon E, Majzoub J, Neupert P, McCallie D, Szolovits P, Willard HF, Mendelsohn NJ, Temme R, Finkel RS, Yum SW, Medne L, Sunyaev SR, Adzhubey I, Cassa CA, de Bakker PI, Duzkale H, Dworzy Ski P, Fairbrother W (2014). An international effort towards developing standards for best practices in analysis, interpretation and reporting of clinical genome sequencing results in the CLARITY Challenge. Genome Biol.

[32] Rehm HL, Bale SJ, Bayrak-Toydemir P, Berg JS, Brown KK, Deignan JL, Friez MJ, Funke BH, Hegde MR, Lyon E (2013). Working Group of the American College of Medical Genetics and Genomics Laboratory Quality Assurance Commitee: ACMG clinical laboratory standards for next-generation sequencing. Genet Med.

[33] Ashley EA, Butte AJ, Wheeler MT, Chen R, Klein TE, Dewey FE, Dudley JT, Ormond KE, Pavlovic A, Morgan AA, Pushkarev D, Neff NF, Hudgins L, Gong L, Hodges LM, Berlin DS, Thorn CF, Sangkuhl K, Hebert JM, Woon M, Sagreiya H, Whaley R, Knowles JW, Chou MF, Thakuria JV, Rosenbaum AM, Zaranek AW, Church GM, Greely HT, Quake SR (2010). Clinical assessment incorporating a personal genome. Lancet.

[34] Sturm AC, Manickam K (2012). Direct-to-consumer personal genomic testing: a case study and practical recommendations for "genomic counseling". J Genet Couns.

[35] Dewey FE, Grove ME, Pan C, Goldstein BA, Bernstein JA, Chaib H, Merker JD, Goldfeder RL, Enns GM, David SP, Pakdaman N, Ormond KE, Caleshu C, Kingham K, Klein TE, Whirl-Carrillo M, Sakamoto K, Wheeler MT, Butte AJ, Ford JM, Boxer L, Ioannidis JP, Yeung AC, Altman RB, Assimes TL, Snyder M, Ashley EA, Quertermous T (2014). Clinical interpretation and implications of whole-genome sequencing. JAMA.

[36] Machini K, Douglas J, Braxton A, Tsipis J, Kramer K (2014). Genetic counselors' views and experiences with the clinical integration of genome sequencing. J Genet Couns.

[37] ᅟ (2008). American College of Medical Genetics and Genomics.

[38] College of American Pathologists: *Molecular Pathology Checklist.* Northfield: ᅟ 2013.

[39] Clinical and Laboratory Standards Institute (2006). Molecular Diagnostic Methods for Genetic Diseases; Approved Guideline.

[40] Clinical and Laboratory Standards Institute: *Verification and Validation of Multiplex Nucleic Acid Assays; Approved Guideline. Wayne.* 2008.

[41] Clinical and Laboratory Standards Institute: *Nucleic Acid Sequencing Methods in Diagnostic Laboratory Medicine; Approved Guideline. Wayne.* 2007.

[42] Gulley ML, Braziel RM, Halling KC, Hsi ED, Kant JA, Nikiforova MN, Nowak JA, Ogino S, Oliveira A, Polesky HF, Silverman L, Tubbs RR, Van Deerlin VM, Vance GH, Versalovic J (2007). Molecular Pathology Resource Committee, College of American Pathologists: Clinical laboratory reports in molecular pathology. Arch Pathol Lab Med.

[43] Organisation for Economic Co-operation and Development: *OECD Guidelines for Quality Assurance in Molecular Genetic Testing. Paris.* 2007.

[44] Chen B, Gagnon M, Shahangian S, Anderson NL, Howerton DA, Boone JD (2009). Good laboratory practices for molecular genetic testing for heritable diseases and conditions. MMWR Recomm Rep.

[45] ᅟ*Health Level Seven International Standards.* http://www.hl7.org/implement/standards/.

[46] Dorschner MO, Amendola LM, Shirts BH, Kiedrowski L, Salama J, Gordon AS, Fullerton SM, Tarczy-Hornoch P, Byers PH, Jarvik GP (2014). Refining the structure and content of clinical genomic reports. Am J Med Genetics Part C.

[47] Acheson LS, Stange KC, Zyzanski S (2005). Clinical genetics issues encountered by family physicians. Genet Med.

[48] Andersson HC, Krousel-Wood MA, Jackson KE, Rice J, Lubin IM (2002). Medical genetic test reporting for cystic fibrosis (deltaF508) and factor V Leiden in North American laboratories. Genet Med.

[49] Giardiello FM, Brensinger JD, Petersen GM, Luce MC, Hylind LM, Bacon JA, Booker SV, Parker RD, Hamilton SR (1997). The use and interpretation of commercial APC gene testing for familial adenomatous polyposis. N Engl J Med.

[50] Lubin IM, Caggana M, Constantin C, Gross SJ, Lyon E, Pagon RA, Trotter TL, Wilson JA, McGovern MM (2008). Ordering molecular genetic tests and reporting results: practices in laboratory and clinical settings. J Mol Diagn.

[51] McGovern MM, Benach M, Zinberg R (2003). Interaction of genetic counselors with molecular genetic testing laboratories: implications for non-geneticist health care providers. Am J Med Genet Part A.

[52] Hammond EH, Flinner RL (1997). Clinically relevant breast cancer reporting: using process measures to improve anatomic pathology reporting. Arch Pathol Lab Med.

[53] Laposata ME, Laposata M, Van Cott EM, Buchner DS, Kashalo MS, Dighe AS (2004). Physician survey of a laboratory medicine interpretive service and evaluation of the influence of interpretations on laboratory test ordering. Arch Pathol Lab Med.

[54] Lubin IM, McGovern MM, Gibson Z, Gross SJ, Lyon E, Pagon RA, Pratt VM, Rashid J, Shaw C, Stoddard L, Trotter TL, Williams MS, Amos Wilson J, Pass K (2009). Clinician perspectives about molecular genetic testing for heritable conditions and development of a clinician-friendly laboratory report. J Mol Diagn.

[55] Shirts BH, Larsen N, Jackson BR (2012). Utilization and utility of clinical laboratory reports with graphical elements. J Pathol Inform.

[56] Butow PN, Lobb EA (2004). Analyzing the process and content of genetic counseling in familial breast cancer consultations. J Genet Couns.

[57] Cameron LD, Marteau TM, Brown PM, Klein WM, Sherman KA (2012). Communication strategies for enhancing understanding of the behavioral implications of genetic and biomarker tests for disease risk: the role of coherence. J Behav Med.

[58] Contegiacomo A, Pensabene M, Capuano I, Tauchmanova L, Federico M, Turchetti D, Cortesi L, Marchetti P, Ricevuto E, Cianci G, Venuta S, Barbieri V, Silingardi V (2004). Italian Network on Hereditary Breast Cancer: An oncologist-based model of cancer genetic counselling for hereditary breast and ovarian cancer. Ann Oncol.

[59] Croyle RT, Lerman C (1999). Risk communication in genetic testing for cancer susceptibility. J Natl Cancer Inst Monogr.

[60] Daly MB, Barsevick A, Miller SM, Buckman R, Costalas J, Montgomery S, Bingler R (2001). Communicating genetic test results to the family: a six-step, skills-building strategy. Fam Community Health.

[61] Pieterse AH, van Dulmen S, van Dijk S, Bensing JM, Ausems MG (2006). Risk communication in completed series of breast cancer genetic counseling visits. Genet Med.

[62] Centers for Medicare and Medicaid Services: *Eligible Professional Meaningful Use Core Measures.* **Measure 7 of 17.** [http://www.cms.gov/Regulations-and-Guidance/Legislation/EHRIncentivePrograms/downloads/Stage2_EPCore_7_PatientElectronicAccess.pdf].

[63] DHHS: **45 CFR Part 170. Health and information technology standards, implementation specifications, and certification criteria for electronic health record technology.** *Fed Regist* 2012, **77:**54163. 22946139

[64] Archer N, Fevrier-Thomas U, Lokker C, McKibbon KA, Straus SE (2011). Personal health records: a scoping review. J Am Med Inform Assoc.

[65] Centers for Medicare and Medicaid Services, Centers for Disease Control and Prevention, Office for Civil Rights (2014). CLIA program and HIPAA privacy rule; patients' access to test reports. Final rule Fed Regist.

[66] Priyanath A, Feinglass J, Dolan NC, Haviley C, Venta LA (2002). Patient satisfaction with the communication of mammographic results before and after the Mammography Quality Standards Reauthorization Act of 1998. AJR Am J Roentgenol.

[67] Ferrante Di Ruffano L, Hyde CJ, McCaffery KJ, Bossuyt PM, Deeks JJ (2012). Assessing the value of diagnostic tests: a framework for designing and evaluating trials. BMJ.

[68] Ad Hoc Committee on Health Literacy for the Council on Scientific Affairs AMA (1999). Health literacy: report of the Council on Scientific Affairs. JAMA.

[69] Lillie SE, Brewer NT, O'Neill SC, Morrill EF, Dees EC, Carey LA, Rimer BK (2007). Retention and use of breast cancer recurrence risk information from genomic tests: the role of health literacy. Cancer Epidemiol Biomarkers Prev.

[70] Brewer NT, Tzeng JP, Lillie SE, Edwards AS, Peppercorn JM, Rimer BK (2009). Health literacy and cancer risk perception: implications for genomic risk communication. Med Decis Making.

[71] Lea DH, Kaphingst KA, Bowen D, Lipkus I, Hadley DW (2011). Communicating genetic and genomic information: health literacy and numeracy considerations. Public Health Genomics.

[72] Gribble JN (1999). Informed consent documents for BRCA1 and BRCA2 screening: how large is the readability gap?. Pat Educ Couns.

[73] Helitzer D, Hollis C, Cotner J, Oestreicher N (2009). Health literacy demands of written health information materials: an assessment of cervical cancer prevention materials. Cancer Control.

[74] McCray AT (2005). Promoting health literacy. J Am Med Inform Assoc.

[75] Alexander RE (2000). Readability of published dental educational materials. J Am Dent Assoc.

[76] Freda MC (2005). The readability of American Academy of Pediatrics patient education brochures. J Pediatr Health Care.

[77] Hoffmann T, McKenna K (2006). Analysis of stroke patients' and carers' reading ability and the content and design of written materials: recommendations for improving written stroke information. Pat Educ Couns.

[78] Cavanaugh K, Huizinga MM, Wallston KA, Gebretsadik T, Shintani A, Davis D, Gregory RP, Fuchs L, Malone R, Cherrington A, Pignone M, DeWalt DA, Elasy TA, Rothman RL (2008). Association of numeracy and diabetes control. Ann Intern Med.

[79] Osborn CY, Cavanaugh K, Wallston KA, White RO, Rothman RL (2009). Diabetes numeracy mediates the association between African American race and poor glycemic control. Diabetes Care.

[80] Henneman L, Marteau TM, Timmermans DR (2008). Clinical geneticists' and genetic counselors' views on the communication of genetic risks: a qualitative study. Pat Educ Couns.

[81] Trevena LJ, Zikmund-Fisher BJ, Edwards A, Gaissmaier W, Galesic M, Han PK, King J, Lawson ML, Linder SK, Lipkus I, Ozanne E, Peters E, Timmermans D, Woloshin S (2013). Presenting quantitative information about decision outcomes: a risk communication primer for patient decision aid developers. BMC Med Inform Decis Mak.

[82] Wick JY (2013). Checking for comprehension: mastering teach-back techniques. Consult Pharm.

[83] Witteman HO, Fuhrel-Forbis A, Wijeysundera HC, Exe N, Dickson M, Holtzman L, Kahn VC, Zikmund-Fisher BJ (2014). Animated randomness, avatars, movement, and personalization in risk graphics. J Med Internet Res.

[84] Bowling BV, Acra EE, Wang L, Myers MF, Dean GE, Markle GC, Moskalik CL, Huether CA (2008). Development and evaluation of a genetics literacy assessment instrument for undergraduates. Genetics.

[85] Saukko PM, Ellard S, Richards SH, Shepherd MH, Campbell JL (2007). Patients' understanding of genetic susceptibility testing in mainstream medicine: qualitative study on thrombophilia. BMC Health Serv Res.

[86] Syurina EV, Brankovic I, Probst-Hensch N, Brand A (2011). Genome-based health literacy: a new challenge for public health genomics. Public Health Genomics.

[87] Vassy JL, O'Brien KE, Waxler JL, Park ER, Delahanty LM, Florez JC, Meigs JB, Grant RW (2012). Impact of literacy and numeracy on motivation for behavior change after diabetes genetic risk testing. Med Decis Making.

[88] Goos LM, Silverman I, Steele L, Stockley T, Ray PN (2004). Providing information at the point of care: educational diagnostic reports from a genetic testing service provider. Clin Leadersh Manag Rev.

[89] Norman CD, Skinner HA (2006). eHealth literacy: essential skills for consumer health in a networked world. J Med Internet Res.

[90] Hassol A, Walker JM, Kidder D, Rokita K, Young D, Pierdon S, Deitz D, Kuck S, Ortiz E (2004). Patient experiences and attitudes about access to a patient electronic health care record and linked web messaging. J Am Med Inform Assoc.

[91] Matheny ME, Gandhi TK, Orav EJ, Ladak-Merchant Z, Bates DW, Kuperman GJ, Poon EG (2007). Impact of an automated test results management system on patients' satisfaction about test result communication. Arch Intern Med.

[92] Bernhardt JM, Lariscy RA, Parrott RL, Silk KJ, Felter EM (2002). Perceived barriers to Internet-based health communication on human genetics. J Health Commun.

[93] Green MJ, Biesecker BB, McInerney AM, Mauger D, Fost N (2001). An interactive computer program can effectively educate patients about genetic testing for breast cancer susceptibility. Am J Med Genet.

[94] Green MJ, Peterson SK, Baker MW, Harper GR, Friedman LC, Rubinstein WS, Mauger DT (2004). Effect of a computer-based decision aid on knowledge, perceptions, and intentions about genetic testing for breast cancer susceptibility: a randomized controlled trial. JAMA.

[95] Bernhardt JM, McClain J, Parrott RL (2004). Online health communication about human genetics: perceptions and preferences of internet users. Cyberpsychol Behav.

[96] Croyle RT, Lerman C (1999). Risk communication in genetic testing for cancer susceptibility. J Natl Cancer Inst Monogr.

[97] Aktan-Collan K, Haukkala A, Mecklin JP, Uutela A, Kaariainen H (2001). Comprehension of cancer risk one and 12 months after predictive genetic testing for hereditary non-polyposis colorectal cancer. J Med Genet.

[98] Miller SM, Bowen DJ, Campbell MK, Diefenbach MA, Gritz ER, Jacobsen PB, Stefanek M, Fang CY, Lazovich D, Sherman KA, Wang C (2004). Current research promises and challenges in behavioral oncology: report from the American Society of Preventive Oncology annual meeting, 2002. Cancer Epidemiol Biomarkers Prev.

[99] Petty RE, Wegener DT, Fabrigar LR (1997). Attitudes and attitude change. Annu Rev Psychol.

[100] Etchegary H, Perrier C (2007). Information processing in the context of genetic risk: implications for genetic-risk communication. J Genet Couns.

[101] DiLorenzo T, Schnur J, Montgomery GH, Erblich J, Winkel G, Bovbjerg DH (2006). A model of disease-specific worry in heritable disease: the influence of family history, perceived risk, and worry about other illnesses. J Behav Medic.

[102] LaRusse S, Roberts JS, Marteau TM, Katzen H, Linnenbringer EL, Barber M, Whitehouse P, Quaid K, Brown T, Green RC, Relkin NR (2005). Genetic susceptibility testing versus family history-based risk assessment: Impact on perceived risk of Alzheimer disease. Genet Med.

[103] Marteau TM, Roberts S, LaRusse S, Green RC (2005). Predictive genetic testing for Alzheimer's disease: impact upon risk perception. Risk Anals.

[104] Catz DS, Green NS, Tobin JN, Lloyd-Puryear MA, Kyler P, Umemoto A, Cernoch J, Brown R, Wolman F (2005). Attitudes about genetics in underserved, culturally diverse populations. Community Genet.

[105] Marteau TM, Weinman J (2006). Self-regulation and the behavioural response to DNA risk information: a theoretical analysis and framework for future research. Soc Sci Med.

[106] Brewer NT, Weinstein ND, Cuite CL, Herrington JE (2004). Risk perceptions and their relation to risk behavior. Ann Behav Med.

[107] van der Pligt J (1998). Perceived risk and vulnerability as predictors of precautionary behaviour. Brit J Health Psych.

[108] Valenstein PN (2008). Formatting pathology reports: applying four design principles to improve communication and patient safety. Arch Pathol Lab Med.

[109] Meilleur KG, Littleton-Kearney MT (2009). Interventions to improve patient education regarding multifactorial genetic conditions: a systematic review. Am J Med Genet A.

[110] Lewis C, Mehta P, Kent A, Skirton H, Coviello D (2007). An assessment of written patient information provided at the genetic clinic and relating to genetic testing in seven European countries. Eur J Hum Genet.

[111] Amado RG, Wolf M, Peeters M, Van Cutsem E, Siena S, Freeman DJ, Juan T, Sikorski R, Suggs S, Radinsky R, Patterson SD, Chang DD (2008). Wild-type KRAS is required for panitumumab efficacy in patients with metastatic colorectal cancer. J Clin Oncol.

[112] Benesova L, Minarik M, Jancarikova D, Belsanova B, Pesek M (2010). Multiplicity of EGFR and KRAS mutations in non-small cell lung cancer (NSCLC) patients treated with tyrosine kinase inhibitors. Anticancer Res.

[113] De Roock W, Jonker DJ, Di Nicolantonio F, Sartore-Bianchi A, Tu D, Siena S, Lamba S, Arena S, Frattini M, Piessevaux H, Van Cutsem E, O'Callaghan CJ, Khambata-Ford S, Zalcberg JR, Simes J, Karapetis CS, Bardelli A, Tejpar S (2010). Association of KRAS p.G13D mutation with outcome in patients with chemotherapy-refractory metastatic colorectal cancer treated with cetuximab. JAMA.

[114] Kincaid J, Fishburne RP Jr, Rogers RL, Chissom BS: ***Derivation of new readability formulas (Automated Readability Index, Fog Count and Flesch Reading Ease Formula) for Navy enlisted personnel.*** *Research Branch Report 8–75.* Millington TN: Naval Technical Training US Naval Air Station, Memphis, TN; 1975.

[115] Baker DL, Eash T, Schuette JL, Uhlmann WR (2002). Guidelines for writing letters to patients. J Genet Couns.

[116] Green N (2005). GenIE: an intelligent system for writing genetic counseling patient letters. AMIA Annu Symp Proc.

[117] Green N, Dwight R, Navoraphan K, Stadler B (2011). Natural language generation of transparent arguments for lay audiences. Arg Comp.

[118] Green NL, Stadler B (2013). Adding coping-related strategies to biomedical argumentation in computer-generated genetic counseling patient letters. Patient Educ Couns.

[119] Roberts NJ, Partridge MR (2006). How useful are post consultation letters to patients?. BMC Med.

[120] Todhunter SL, Clamp PJ, Gillett S, Pothier DD (2010). Readability of out-patient letters copied to patients: can patients understand what is written about them?. J Laryngol Otol.

[121] Barker KL, Reid M, Minns Lowe CJ (2014). What does the language we use about arthritis mean to people who have osteoarthritis? A qualitative study. Disabil Rehabil.

[122] Farrell MH, Christopher SA (2013). Frequency of high-quality communication behaviors used by primary care providers of heterozygous infants after newborn screening. Patient Educ Couns.

[123] Hoffman JM, Haidar CE, Wilkinson MR, Crews KR, Baker DK, Kornegay NM, Yang W, Pui CH, Reiss UM, Gaur AH, Howard SC, Evans WE, Broeckel U, Relling MV (2014). PG4KDS: A model for the clinical implementation of pre-emptive pharmacogenetics. Am J Med Genet C Semin Med Genet.

[124] Polepalli Ramesh B, Houston T, Brandt C, Fang H, Yu H (2013). Improving patients' electronic health record comprehension with NoteAid. Stud Health Technol Inform.

[125] Slaughter L, Oyri K, Fosse E (2011). Evaluation of a Hyperlinked Consumer Health Dictionary for reading EHR notes. Stud Health Technol Inform.

[126] ᅟ*Talking Glossary of Genetic Terms.* http://www.genome.gov/Glossary/.

[127] Brown CE, Roberts NJ, Partridge MR (2007). Does the use of a glossary aid patient understanding of the letters sent to their general practitioner?. Clin Med.

[128] Zeng QT, Tse T, Divita G, Keselman A, Crowell J, Browne AC, Goryachev S, Ngo L (2007). Term identification methods for consumer health vocabulary development. J Med Internet Res.

[129] *How to Write Easy-to-Read Health Materials.* http://www.nlm.nih.gov/medlineplus/etr.html.

[130] Zeng QT, Tse T (2006). Exploring and developing consumer health vocabularies. J Am Med Inform Assoc.

[131] West SL, Squiers LB, McCormack L, Southwell BG, Brouwer ES, Ashok M, Lux L, Boudewyns V, O'Donoghue A, Sullivan HW (2013). Communicating quantitative risks and benefits in promotional prescription drug labeling or print advertising. Pharmacoepidemiol Drug Saf.

[132] Feldman-Stewart D, Kocovski N, McConnell BA, Brundage MD, Mackillop WJ (2000). Perception of quantitative information for treatment decisions. Med Decis Making.

[133] Schapira MM, Nattinger AB, McAuliffe TL (2006). The influence of graphic format on breast cancer risk communication. J Health Commun.

[134] Brewer NT, Gilkey MB, Lillie SE, Hesse BW, Sheridan SL (2012). Tables or bar graphs? Presenting test results in electronic medical records. Med Decis Making.

